# Design of Thermo-Responsive Pervaporation Membrane Based on Hyperbranched Polyglycerols and Elastin-like Protein Conjugates

**DOI:** 10.3390/nano14221821

**Published:** 2024-11-14

**Authors:** Juliet Kallon, John J. Bang, Ufana Riaz, Darlene K. Taylor

**Affiliations:** 1 Department of Pharmaceutical Sciences, North Carolina Central University, Durham, NC 27707, USAjjbang@nccu.edu (J.J.B.); 2 Department of Environmental, Earth and Geospatial Sciences, North Carolina Central University, Durham, NC 27707, USA; 3 Department of Chemistry, North Carolina Central University, Durham, NC 27707, USA

**Keywords:** thermoresponsive polymers, crystal violet, membrane permeability, polymer

## Abstract

This paper reports the development of a highly crosslinked hyper-branched polyglycerol (HPG) polymer bound to elastin-like proteins (ELPs) to create a membrane that undergoes a distinct closed-to-open permeation transition at 32 °C. The crosslinked HPG forms a robust, mesoporous structure (150–300 nm pores), suitable for selective filtration. The membranes were characterized by FTIR, UV–visible spectroscopy, SEM, and AFM, revealing their structural and morphological properties. Incorporating a synthetic polypeptide introduced thermo-responsive behavior, with the membrane transitioning from impermeable to permeable above the lower critical solution temperature (LCST) of 32 °C. Permeation studies using crystal violet (CV) demonstrated selective transport, where CV permeated only above 32 °C, while water permeated at all temperatures. This hybrid HPG-ELP membrane system, acting as a molecular switch, offers potential for applications in drug delivery, bioseparations, and smart filtration systems, where permeability can be controlled by temperature.

## 1. Introduction

Eliminating colorants from wastewater continues to be a significant challenge. This issue partly arises from the collective actions of the industrial, agricultural, and consumer sectors, which release substantial quantities of colored effluent, leading to water contamination. Dye-contaminated water not only lacks aesthetic appeal but also poses significant environmental and long-term human health risks [1]. Among cationic pollutants, crystal violet—commonly used in biological staining, dermatology, and veterinary medicine—is notably more toxic than its anionic counterparts. To decrease the concentration of such contaminants in water to levels that comply with environmental regulations, an interdisciplinary research approach is essential to develop cost-effective and sustainable treatment processes [2,3]. Membrane-based separation and adsorption are two purification techniques that have gained prominence for the removal of dyes from wastewater [4,5,6]. Challenges in developing membrane materials include reducing production costs, incorporating self-cleaning pores for reuse, and ensuring biocompatible properties to minimize secondary contamination [7]. Another approach could involve developing an adsorbent that functions under selective external stimuli, such as mild heat.

Hyperbranched polyglycerols (HPGs) are employed in the development of porous membranes for separation applications. The growing interest in HPG chemistry is largely driven by the polymer’s inherent biocompatibility and the versatility of its derivatives [8]. Furthermore, HPGs exhibit high flexibility due to their aliphatic backbone and the presence of functional hydroxyl groups at their branched ends.

Improvements in the synthetic methodology of these inexpensive and well-defined hyperbranched polymers are receiving increased attention in biomaterial-based applications [9,10]. HPG is prepared in a one-step process, and to endow the membrane with better mechanical strength and solvent resistance properties, these polymers are usually crosslinked [11,12].

Crosslinked hyperbranched polyglycerols (HPGs) create a rigid, mesoporous network, making them excellent candidates for membrane applications. Their inherent hydrophilicity, coupled with hydroxyl functional groups, enhances their suitability for hydrogel design. One notable advantage of these crosslinked materials is the low viscosity of their hydrogel precursors in water [13]. Such polymers have shown promise in adsorption and controlled release of pollutants, both as standalone components and when combined with oxide–silica particles and polyacrylic acid [14,15,16]. However, adsorbents based on this polymer type with self-cleaning pore capabilities have not yet been developed. This functionality could be achieved by integrating the benefits of this platform with smart materials like elastin-like peptides (ELPs).

ELPs are synthetic polypeptides comprising repeating units of the pentapeptide Val-Pro-Gly-Xaa-Gly, where the “guest residue” Xaa is any amino acid except proline [17]. Elastin-like polypeptides (ELPs) are synthetic polypeptides inspired by the sequence and properties of human elastin. While ELPs are often produced using *E. coli* as a recombinant host for expression, they are not derived from *E. coli* itself. Instead, they are engineered polypeptides designed to mimic the thermo-responsive behavior of natural elastin.

ELPs have been used in previous studies for fluid control and molecular transport in biological as well as medical materials, drug delivery devices, and micro-fluidic systems [18]. Porous silica films impregnated with ELPs have been shown to display thermo-responsive behavior acting like memetic switches that open and close in response to temperature changes [18,19]. Costa et al. [20] prepared smart thin coatings using a recombinant elastin-like polymer (ELP) that incorporated the cell attachment sequence arginine–glycine–aspartic acid (RGD) achieved by a process involving ELP dissolved in aqueous solutions. The results showed that the thermo-responsive behavior could be utilized for tunable cell adhesion and controlled protein adsorption. Kim et al. [21] designed thermos-responsive PEGylated human α-elastin, which showed sol-to-particle transition at a lower critical solution temperature (LCST) of 25 °C–40 °C in aqueous media. The ELPs were able to encapsulate significant amounts of insulin and bovine serum albumin (BSA) at a low temperature in water and release profiles of insulin, and BSA showed sustained release for 72 h. Xiao et al. [22] developed a modular approach for creating well-defined block copolymers by combining inert (Dextran) and bioactive (Laminarin and Hyaluronic Acid) polysaccharides with a stimuli-responsive elastin-like polypeptide (ELP), and studied their thermal responsiveness analyzed via turbidity measurements. Lately, Khalili et al. [23] synthesized thermo-responsive biopolymers conjugating ELP functionalized with RGD to a G4 dendrimer as a nanoplatform. Turbidity measurements revealed LCST levels near physiological temperatures, and the polymers showed improved cell adhesion, spreading, proliferation, and differentiation.

The pervaporation process is essentially achieved via the permeability difference between components of a solution permeated across the membrane [12]. Crosslinking of polymers can therefore provide highly selective and improved permeable membranes. Robust membranes with higher selectivity and better permeability than benchmark materials such as solid polymers are still a key consideration in pervaporation [12].

**Scheme 1:**
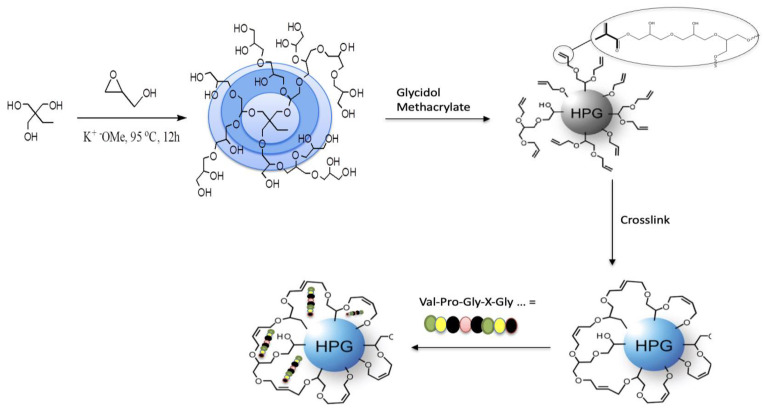
Preparation of functionalized hyperbranched polyglycerols (HPGs) crosslinked to develop pores that incorporate elastin-like proteins.

The present study focuses on the development of a crosslinked HPG polymer that binds an ELP (see Scheme 1). The ELP biomaterials swell in water and typically undergo a phase transition to gel immediately after reaching their Lower Critical Solution Temperature (LCST) [24,25]. In our approach to producing the hybrid membrane, we examined the spectral and morphological properties of ELPs incorporated within crosslinked HPGs. Our investigation demonstrated that this conjugate system has the capability to selectively release crystal violet (CV) in response to changes in temperature.

## 2. Materials and Methods

All chemicals were purchased from Sigma-Aldrich and used as received unless otherwise stated. ELPs were synthesized by the overexpression of a plasmid-borne synthetic gene of the ELP in *Escherichia coli* [21,22,23]. The primary acid sequence of ELP 4-80 (including the leader and trailer functionalization) is Ser-Lys-Gly-Pro-Gly-(Val-Gly-Val-Pro-Gly)_80_-Tyr with an expected molecular weight of 33.37 kDa. The ELP was provided as a gift from Dr. Ashutosh Chilkoti, PhD, Duke University, Durham, N.C., and was stored in lyophilized form at −20 °C until ready for use. Solutions of 100–200 uM ELP 4-80 in ultrapure water were used to perform the temperature-dependent permeation studies.

### 2.1. Synthesis of Hyperbranched Polyglycerol Methacrylate

Hyperbranched polyglycerol (HPG, M_n,MALDI_ = 1096 g mol^−1^, M_w_/M_n_ = 1.13) was synthesized according to the literature by ring-opening polymerization of glycidol at 95 °C for 12 h [10,24]. In a typical experiment, 2.02 g (1.5 × 10^−2^ mol) of 1,1,1-tris(hydroxymethyl)propane (TMP) was 10% deprotonated in an inert atmosphere with sodium methoxide solution (3.7 M in methanol). After removal of the methanol, 50 mL of glycidol was added slowly over 12 h to the stirred melt at a temperature of 95 °C. After workup and characterization, this product was further functionalized with an appropriate amount of glycidyl methacrylate to obtain HPG-MA by the protocol reported by Oudshoorn et al. [13]. Briefly, 4-(N,N-dimethylamino) pyridine, dissolved in dimethyl sulfoxide, catalyzed the reaction of 3 g of HPG with three different concentrations of glycidyl methacrylate (GMA): 5.70 mM, 2.79 mM, and 0.11 mM. The resulting degree of substitution (DS) was confirmed using ^1^H-NMR. The resulting degree of substitution (DS) was confirmed using ^1^H-NMR. The peak areas for the acylate protons associated with the double bonds of methacryloyl groups were integrated and compared to the proton signals of HPG. Based on the percent substitution, the HPG was designated as HPG-28, HPG-15, and HPG-10.

### 2.2. Nuclear Magnetic Resonance

Proton spectra were acquired on a 500 MHz Varian Unity INOVA NMR spectrometer (Varian, Palo Alto, CA, USA) in methyl sulfoxide-d6. Chemical shifts (δ) are reported in parts per million (ppm) referenced to an internal standard of 0.05% TMS. Proton NMR facilitated knowledge about the crosslinking densities and identified the addition of the methyl acrylate groups.

### 2.3. UV–Vis Spectra

Optical measurements were carried out with a Varian Cary 300 UV–Vis Spectrophotometer (Varian, Palo Alto, CA, USA). ELP 4-80 was diluted in phosphate buffer (pH 7.0) solution to two different concentrations of 50 μM or 200 μM. The samples were examined under increasing temperatures ranging from 20 to 60 °C at a heating rate of 1 °C/min. The transition temperature was defined from the heating profile as the temperature at 50% of the maximum turbidity.

### 2.4. Scanning Electron Microscopy

An FEI XL30 SEM-FEG Scanning Electron Microscope (JEOL, Pleasanton, CA, USA) was used to examine the morphology of HPG-28 and HPG-15 films, which were prepared by drop-casting methanol diluted solutions. The films were allowed to air dry for 12–24 h prior to analysis by SEM. As an example of the sample preparation, 0.5 g of the HPG-28 was dissolved in methanol in a 3 × 4 cm circular mold. The sample conformed to the shape of the mold as the methanol was evaporated off. The sample was allowed to air dry for 12 h. A cross-section was obtained by cutting a section of the sample from the mold. The HPG-28 sample was gold sputtered ~4.0 nm thick before being examined by microscopy. The HPG-28 and HPG-15 surface profile and HPG-28 cross-section were specifically examined.

### 2.5. Atomic Force Microscopy (AFM)

The pore sizes and surfaces of HPG-X were measured by tapping-mode AFM (Scanning Microscope Digital Instruments Dimension 3100, Plainview, NY, USA) using a Silicon cantilever. Atomic force micrographs of the HPG-28 and HPG-15 films were studied at ambient temperature conditions, 21 ± 2 °C. The samples were drop-casted on mica disks and left to air dry. Another preparation technique also involved drop-casting the HPG sample on the mica disk, lightly rinsing the excess with water, and lightly drying the films with nitrogen gas.

### 2.6. Crosslinking Procedure

Three distinct substitution degrees of methacrylate-functionalized HPG (HPG-MA; DS = 10, 15 and 28%) were crosslinked chemically with the aid of AD-mix-β similar to the protocol reported by Burakowska et al. [14]. Typically, a 1:1 volume of tert-butyl alcohol and water dissolved 456 mg of AD-mix-beta at room temperature. The mixture was then cooled to 10 °C, and 0.14 g of HPG-MA in 3 mL THF was added, reacting for 20 h.

Crosslinking was also achieved photochemically for the HPG-28-functionalized acrylate sample by following the protocol reported by Oudshoorn et al. [13]. In brief, a phosphate buffer (10 mM, pH 7.2) solution contained 30% (*w*/*v*) of the precursor HPG-MA (DS = 28%) and 0.05% (*w*/*v*) of Irgacure 2959. The solution was coated onto Millipore Micron centrifugal tubes (Sigma Aldrich, St. Louis, MO, USA; diameter, 1.23 cm; active membrane area, 0.32 cm^2^) with 30,000-Da (YM-30) molecular weight cut-off membranes. Coating was enabled with a benchtop centrifuge (Eppendorf Centrifuge 5804 R outfitted with FA-45-6-30 rotor) operating at 310–551 rcf. Afterward, the coated filters were flushed with nitrogen and photopolymerized by exposure to 365 nm UV light at 13,000 mW/cm^2^ (Spectroline 4 UVC Model, Sigma Aldrich, St. Louis, MO, USA) for 7 min. Unreacted materials and the initiator were flushed out with 10–20 mL of 1 M NaOH (in order to hydrolyze the unreacted methacrylate groups) followed by a dimethyl chloride rinse. After room temperature air drying for 12 h, the ELP 4-80 construct solutions (25–200 μM in PBS) were coated onto the HPG-X prepared films and allowed to diffuse overnight at room temperature.

### 2.7. Permeation Studies

Permeation experiments were carried out using Eppendorf 5804R tubes (Enfield, CT, USA) by centrifuging at 1000 rpm for 3 min at a select temperature within the range of 25 to 40 °C. In these experiments, crystal violet (0.1 wt % aqueous solution) was centrifuged through ELP-coated, HPG-coated, HPG-28-coated, and HPG-28/ELP-coated YM-30 centrifugal filters. The prepped filters were equilibrated for 10 min in Isotemp 125D heating blocks at the test temperature prior to transferring them to the temperature equilibrated centrifuge.

ELP 4-80 samples were prepared by dilutions of the sample in PBS to concentrations of 50 and 200 µM. The ELP solution was individually drop-casted and spin coated into the centrifuge filter and left to dry overnight. HPG-28 and ELP + HPG-28 were also individually used to coat the centrifugal filters. An uncoated filter was used as a control for the experiments.

## 3. Results and Discussion

### 3.1. NMR Analysis

**Figure 1:**
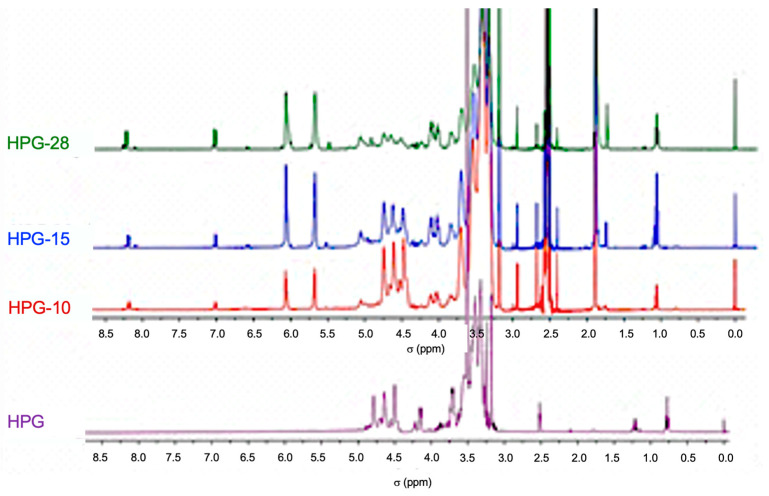
Nuclear magnetic resonance spectra of HPG and methacrylate-functionalized HPG samples. Note the ^1^H NMR spectra of HPG displays no peaks in the 5.5–6.5 ppm, denoting the lack of methacrylate protons in this spectrum. These peaks are present in HPG-10, HPG-15, and HPG-28 ^1^H NMR spectra.

The NMR spectra presented in Figure 1 indicate that the HPG was successfully prepared. Under the specified reaction conditions—room temperature in a nitrogen atmosphere, using DMSO as the solvent and DMAP as the catalyst—the methacryloyl groups were directly linked to the polymer via transesterification [13,14]. The NMR spectrum of HPG shows the methylene protons at 3.4 ppm and the hydroxyl signal at 4.6 ppm. When the glycidol monomer ring opened to add the tris- hydroxyl initiating core, each monomer unit contributed an average of six protons which were all detected at 3.4 ppm. The hydroxyl protons were detected at 4.6 ppm.

Proton NMR determined the relative functionalization of HPG with methyl acrylate groups and thus the upper limit on the respective crosslink density for HPG-28, HPG-15, and HPG-10. Upon the incorporation of the methacryloyl groups, new proton peaks were observed at 1.8 ppm (CH_3_) and at 5.67 ppm and 6.08 ppm attributed to (CH_2_=CH). The integration of the peak areas for these acrylate protons associated with the double bond relative to the HPG protons provided an estimate of the fraction of HPG derivatized with methacrylate groups and thus the yield HPG-MA. After the addition of glycidyl methacrylate, peaks appeared at 5.6 and 6.0 ppm that indicated the addition of methyl acrylate groups. Only HPG-28 and HPG-15 provided quality films to be used in the development of the hydrogel membranes.

### 3.2. UV–VIS Analysis

**Figure 2:**
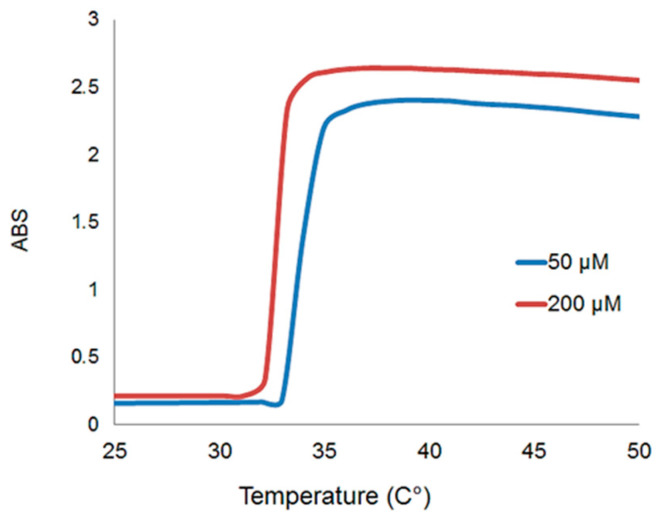
Absorbance of ELP 4-80 as a function of temperature. The UV–Vis spectra show a sharp increase at 33 and 34 °C for 50 µM and 200 µM solutions, respectively, indicating the occurrence of the phase transition.

The ELP transition plays a major role in the membrane function during increasing temperature changes. Understanding the transition temperature of ELP is vital to determining the thermo-responsive properties of the hybrid membrane. An established transition temperature range provides information of when soluble ELP changes its confirmation to an insoluble aggregate. The transition of ELPs from soluble to insoluble in response to environmental stimuli was observed and quantified using UV–Vis spectroscopy. The temperature-induced aggregation of the proteins was characterized by monitoring absorbance at 350 nm as a function of temperature. The transition temperature was defined from the heating profile as the temperature at 50% of the maximum turbidity. These molecular changes were seen to be a gradual process that started at temperatures well below the transition temperature. In response to increasing solution temperature, ELPs exhibit a hydrophobic-to-hydrophilic phase transition when heated, where they transition from a soluble, hydrophilic state below the LCST to an aggregated, hydrophobic state above the LCST [23]. ELPs dissolve well in water at or below room temperature, creating clear and homogenous solutions. However, when the temperature increases above a certain threshold, these solutions undergo a sudden phase transition and turn cloudy. This change, which occurs at the LCST, is due to a temperature-driven conformational shift in the polypeptides. This shift causes the polypeptides to aggregate and lose solvation, resulting in an inverse temperature-dependent liquid−liquid-phase separation, also known as coacervation. The polypeptide aggregates scattered light, causing an increase in the turbidity of the polypeptide solution [26,27]. The UV spectra results shown in Figure 2 confirm that the transition temperature of ELP 4-80 was approximately 33 to 34 °C.

The transition temperature was in accordance with the referenced temperature for this construct [28]. Concentration affected the observed transition temperature where the spectra for both 50 µM and 200 µM samples are shown in Figure 2. For high molecular weight ELP, a higher concentration yielded more defined and stronger transition molecules due to rapid change in turbidity compared to a 50 µM solution of ELP 4-80. At higher concentrations, such as 200 µM, the increased density of ELP molecules led to enhanced intermolecular interactions. This heightened interaction can result in more pronounced hydrophobic interactions, which contribute to a more defined phase transition. As the temperature rises, these interactions facilitate a rapid aggregation of the ELP molecules, which is reflected in a sharper transition temperature. The turbidity of a solution is an indicator of the concentration of suspended particles or aggregates. In this case, the transition from a clear solution to a turbid state signifies the onset of aggregation as the temperature approaches the LCST. The 200 µM ELP solution exhibited a more rapid and significant change in turbidity compared to the 50 µM solution. This rapid change indicates that the molecules in the higher concentration solution are more likely to interact and aggregate quickly as the temperature rises, leading to a stronger and more defined transition. The spectra obtained from both concentrations displayed differences in intensity and shape at the transition temperature. For the higher concentration sample (200 µM), the spectrum showed a sharper peak/distinct change in absorbance due to the greater number of aggregated molecules interacting with light. Conversely, the 50 µM ELP solution showed a less pronounced transition, with a broader spectrum that suggests a more gradual aggregation process.

### 3.3. SEM Analysis

**Figure 3:**
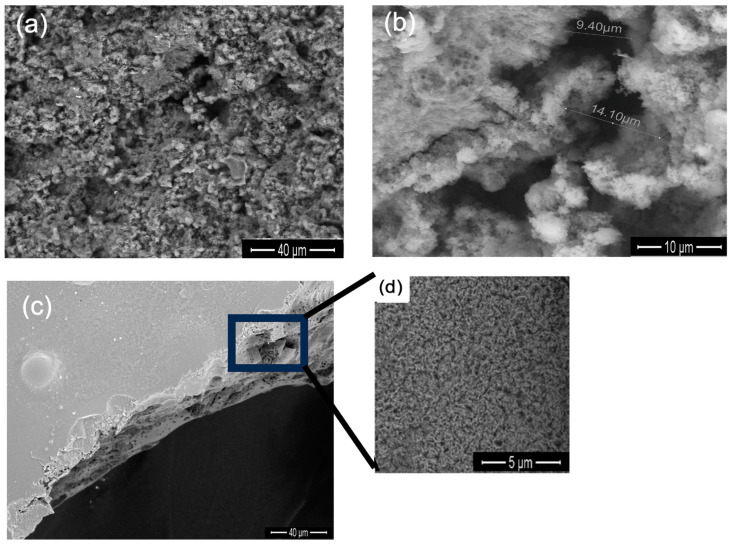
SEM micrographs of HPG-28 lateral cross-sections observed at 40 micron (**a**), cross-sections observed at 10 micron (**b**), and edge side view when mixed with 200 µM ELP 4-80 (**c**). A magnified view of edge of the ELP + HPG-28 membrane reveals less distinct pores compared to HPG-28 alone, giving the appearance that the ELP chain has occupied the main structure of the pores created by HPG-28. (**d**) Enlargment of cross section shows a uniform and solid, homogenous structure.

A range of pore sizes were confirmed for the HPG membranes in both the medium and high crosslinked structures (see Figure S1). Figure 3 displays a cross-section of HPG-28 and HPG-15 membranes. The SEM images display a thin and nonhomogeneous mixture in both cases with a range of nano pore sizes. However, the pores were more distinct in HPG-28, Figure 3a than those observed in HPG-15, Figure 3b. The structural integrity of crosslinked films increases with the formation of crosslinking bridges. To demonstrate the conjugate membrane morphology, SEM micrographs for the internal structures of the HPG-28 with and without ELP were observed. Enhanced magnification of HPG-28 is presented in Figure 3a. The crosslinked HPG-28 displayed pore sizes that range from approximately <15 to 20 nm. Evidence of the appearance of a network of various pores and channels below the polymer surface was clearly noted. Large macro voids in the substructure typically resulted in increased permeability, and therefore, may be desirable in moderate-pressure Ultra Filtration applications such as in dialysis and downstream processing [28,29]. These observations confirmed that the HPG was successfully crosslinked and had formed a large network of pores. This dense morphology was essential for the crosslinked HPG membranes to be considered for a pervaporation process [11]. Along with the morphologies, SEM also ensured that the membrane produced a defect-free film for permeation studies.

### 3.4. AFM

**Figure 4:**
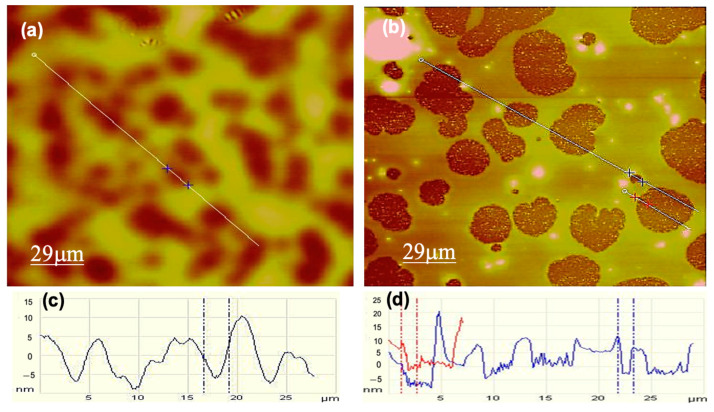
AFM images on mica of (**a**) HPG-28 and (**b**) HPG-15 (experimental repeats, n = 3) membranes. Height distribution of pores for (**c**) HPG-28 and (**d**) HPG-15.

The AFM images of HPG-28 films shown in Figure 4a,b also confirm the existence of a range of pore sizes. The images are representative and show 3.5 μ and 6 μ pores of HPG-28. The spherical particles on the HPG surface confirm the existence of complex HPG surface structures, like the “bead-on-string” morphology exhibited in highly crosslinked polymer films. The molecular ordering of the crosslinked HPG represents the key to the nanophase separation and the in-situ formation of nanocoating composed of nanolayer superstructures [21]. These results are consistent with other films where moderate conglomerates of spherical particles with bead-on-string morphology were formed [26,30]. The pore range of the HPG-28, HPG-15, and HPG-10 is summarized in Table 1, and together offer a very broad range of sizes that can enhance selectivity.

However, micron-sized pores could also be a disadvantage and their formation is important in developing membranes. The broader range of pores observed in HPG-28 relative to HPG-15 provides the best quality framework in the development of the conjugate HPG-X/ELP membrane system. Once confirmation of the individual characteristics of the HPG-X and ELP were obtained, the conjugate hydrogel membranes were formed.

### 3.5. Permeation 

**Figure 5:**
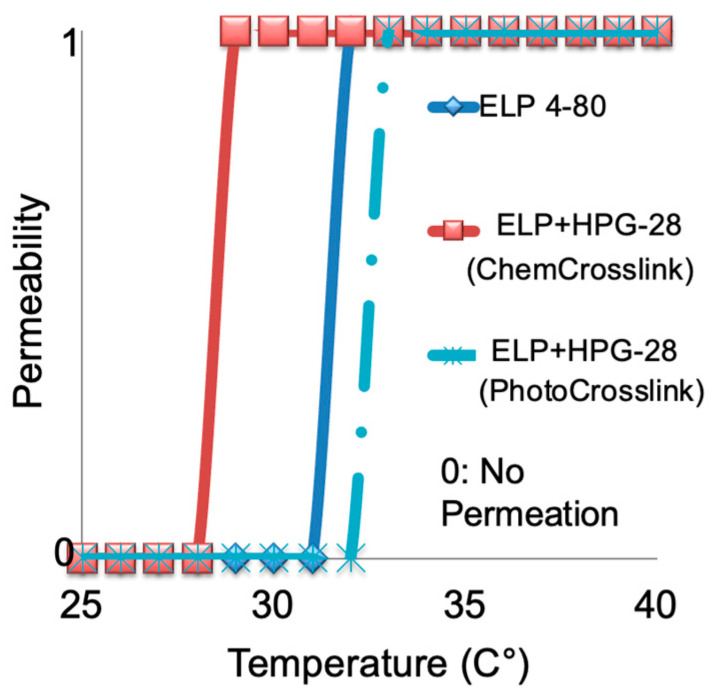
Permeation of crystal violet solution (400 μL) through ELP 4-80, ELP + HPG-28 chemically crosslinked, ELP + HPG-28 photochemically crosslinked membranes. “No permeation” denotes that no water was observed to permeate through the filter. Each data point was obtained after 3 min of centrifugation at the indicated centrifugal force.

Figure 5 illustrates the permeation of crystal violet (CV) through ELP-, ELP + HPG-28-, and HPG-28-coated filters using temperature-controlled centrifugation. HPG crosslinked membranes were used as a control and were shown to be permeable to CV solutions at all temperatures. In contrast, the crosslinked HPG-28 membranes remained impermeable to CV solutions across all temperature ranges. However, the conjugate membranes exhibited selective permeation. When the temperature was below 30 °C (under the lower critical solution temperature (LCST) of ELP), the membranes were impermeable to aqueous solutions. Above 30 °C, the conjugate membranes became permeable to the CV solution. Notably, above the LCST, the hybrid membrane and ELP membrane behaved similarly in terms of CV permeation, although the hybrid-coated membrane demonstrated a temperature transition shift compared to the ELP sample. These results are corroborated with photographs of filtrate (or lack of filtrate) as a function of temperature after permeation studies with the ELP-, ELP + HPG-28-, and HPG-28-coated filters (see Figure S2).

### 3.6. Advantages of Designing ELP-HPG Membranes

While some thermos-responsive materials rely solely on synthetic polymers, the integration of ELPs allows the membrane to leverage natural phase-transition properties that offer a more predictable and precise temperature response. The mesoporous network provides a balance between mechanical strength and flexibility, which may outperform existing membranes that either lack robustness or have limited adaptability. The simplicity of UV crosslinking and the cost-effective nature of combining HPGs with ELPs suggest a potential reduction in production costs compared to more complex membrane systems. Additionally, the self-regulating permeability could lower operational costs in filtration and separation processes by minimizing energy consumption associated with active temperature control. By using biocompatible and less chemically intensive materials, the production process of HPG-ELP membranes could result in fewer environmental pollutants. The self-cleaning nature of the membrane could also reduce the frequency of replacements and maintenance, decreasing waste and promoting sustainable use. Furthermore, the temperature-responsive functionality aligns with efforts to create energy-efficient systems, as they utilize natural phase changes rather than mechanical or electrical means for switching permeability states.

## 4. Conclusions

A membrane system using crosslinked HPG-28 in combination with elastin-like proteins (ELPs) was successfully developed. The crosslinking of HPG created a robust, mesoporous network, which provides structural integrity and enhanced stability to the membrane. Scanning Electron Microscopy (SEM) analysis revealed a variety of pore sizes within the HPG-28 structure and insights about how ELPs incorporate within the HPG framework to form hybrid membranes. The combination of HPGs and ELPs created a unique membrane capable of functioning as a molecular thermo-responsive switch, where the permeability was controlled by the phase transitions of the ELPs. The hybrid membranes exhibited selective permeability based on temperature, with ELPs acting as a trigger. Below a certain temperature, the membranes remained impermeable, while at higher temperatures, the phase transition of the ELPs allowed permeability. This dynamic behavior allowed the hybrid membrane to act as a nano-molecular valve. The integration of these materials into a single membrane system offers potential for applications that require controlled, responsive permeation. Future scope of work: While the development of the membrane system combining crosslinked HPG-28 with elastin-like proteins (ELPs) demonstrates promising results, there are several areas for future research and development to further enhance the capabilities and applications of these materials. Further research could focus on fine-tuning the pore size distribution within the HPG-28 network to improve selectivity and permeability for specific applications, such as targeted molecule filtration or controlled drug delivery. Investigating methods to enhance the long-term stability and mechanical strength of the membranes, especially under harsh environmental or operational conditions, would be critical for broader industrial applications. While the current system leverages thermo-responsive properties of ELPs, future work could explore additional stimuli, such as pH, light, or specific ions, to create more versatile membranes capable of responding to diverse environmental triggers. Expanding the investigation into the biocompatibility and safety of these membranes for biomedical applications, such as tissue engineering, wound healing, or drug delivery, would be a significant area for further research. By addressing these concerns, future work on HPG-28 ELP hybrid membranes could expand their applicability across a broad range of fields, from environmental science to biotechnology and industrial processing. Technological significance: Technologically, these hybrid membranes are expected to offer significant advancements in separation technologies by providing a cost-effective, temperature-responsive alternative to conventional systems. Applications in biotechnology could revolutionize areas like controlled drug delivery, where temperature-triggered release ensures targeted and precise administration of therapeutics. Furthermore, the potential to integrate additional stimuli responses, such as pH and light, could lead to the development of multi-functional membranes suitable for diverse uses, including environmental monitoring and advanced water purification. In summary, the advancement of crosslinked HPG-ELP hybrid membranes has the potential to drive forward both scientific exploration and technological innovation, impacting fields ranging from industrial processing to medical devices and environmental engineering.

## Figures and Tables

**Table 1:** Summary of crosslinked HPG:X samples characterized by ^1^H NMR, AFM, and SEM and polymerization.

HPG-X	GMA (mM)	Degree of Crosslink (^1^H NMR)	Appearance	Pore Size (AFM)	Pore Size (SEM)
HPG-28	0.111	28%	White, solid	3–12 mm	1–30 mm
HPG-15	0.054	15%	Sticky solid	6–20 mm	20–30 mm
HPG-10	0.022	10%	Viscous film	indistinguishable	indistinguishable

## Data Availability

Data are included in the manuscript and Supporting Information.

## Supplementary Materials

The following supporting information can be downloaded at: https://www.mdpi.com/article/10.3390/nano14221821/s1, Figure S1: Surface of HPG based membranes. SEM micro-graphs show HPG-28 surface at 20 micron scale (A) and 3 micron scale. SEM micrographs of HPG-15 are shown at 20 micron (C) and 1 micron (D). Figure S2. Permeation experiment of crystal violet. Photos of the solutions after filtration through YM-30 uncoated (control) or coated mem-branes with one of three surface treatments: ELP 4-80, HPG-28, or ELP with HPG-28. The permeation tests were evaluated at four different temperatures: 25 °C, 28 °C, 33 °C, and 37 °C.
