# Supplementary material for: Design of Thermo-Responsive Pervaporation Membrane Based on Hyperbranched Polyglycerols and Elastin-like Protein Conjugates
Source: Nanomaterials (Basel). 2024 Nov 14;14(22):1821. doi: 10.3390/nano14221821 (PMC11597402; doi:10.3390/nano14221821)
Supplement: Supplementary file 1 [file nanomaterials-14-01821-s001.zip › nanomaterials-3290683-supplementary.pdf]

Supporting Information

Design of Thermo-Responsive Pervaporation Membrane Based on Hyperbranched Polyglycerols and Elastin-like Protein Conjugates

Juliet Kallon [1], John J. Bang [1,2], Ufana Riaz [3] and Darlene K. Taylor [1,3,*]

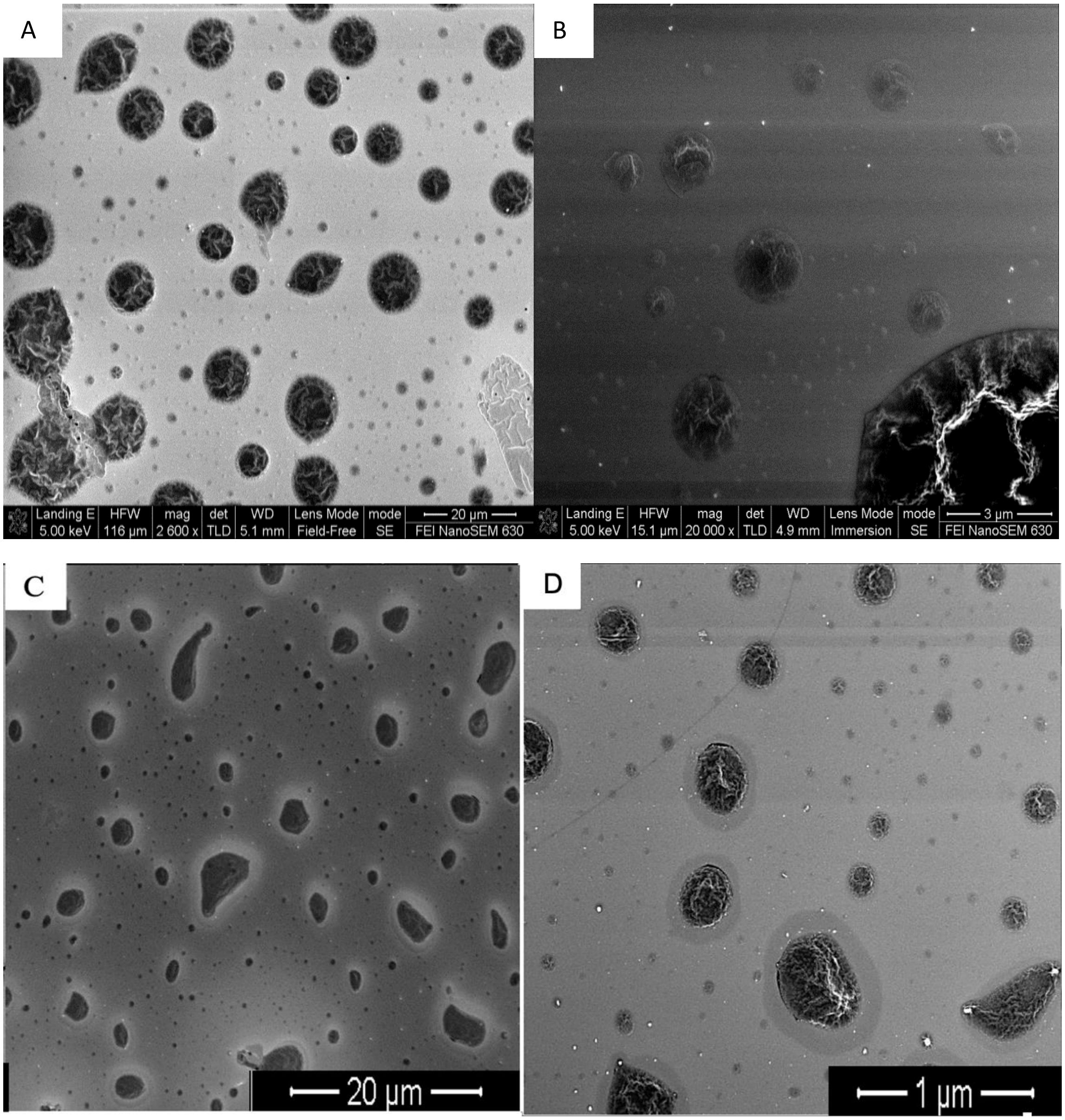

Figure S1. Surface of HPG based membranes. SEM micrographs show HPG-28 surface at 20 micron scale (A) and 3 micron scale (B). SEM micrographs of HPG-15 are shown at 20 micron (C) and 1 micron (D).

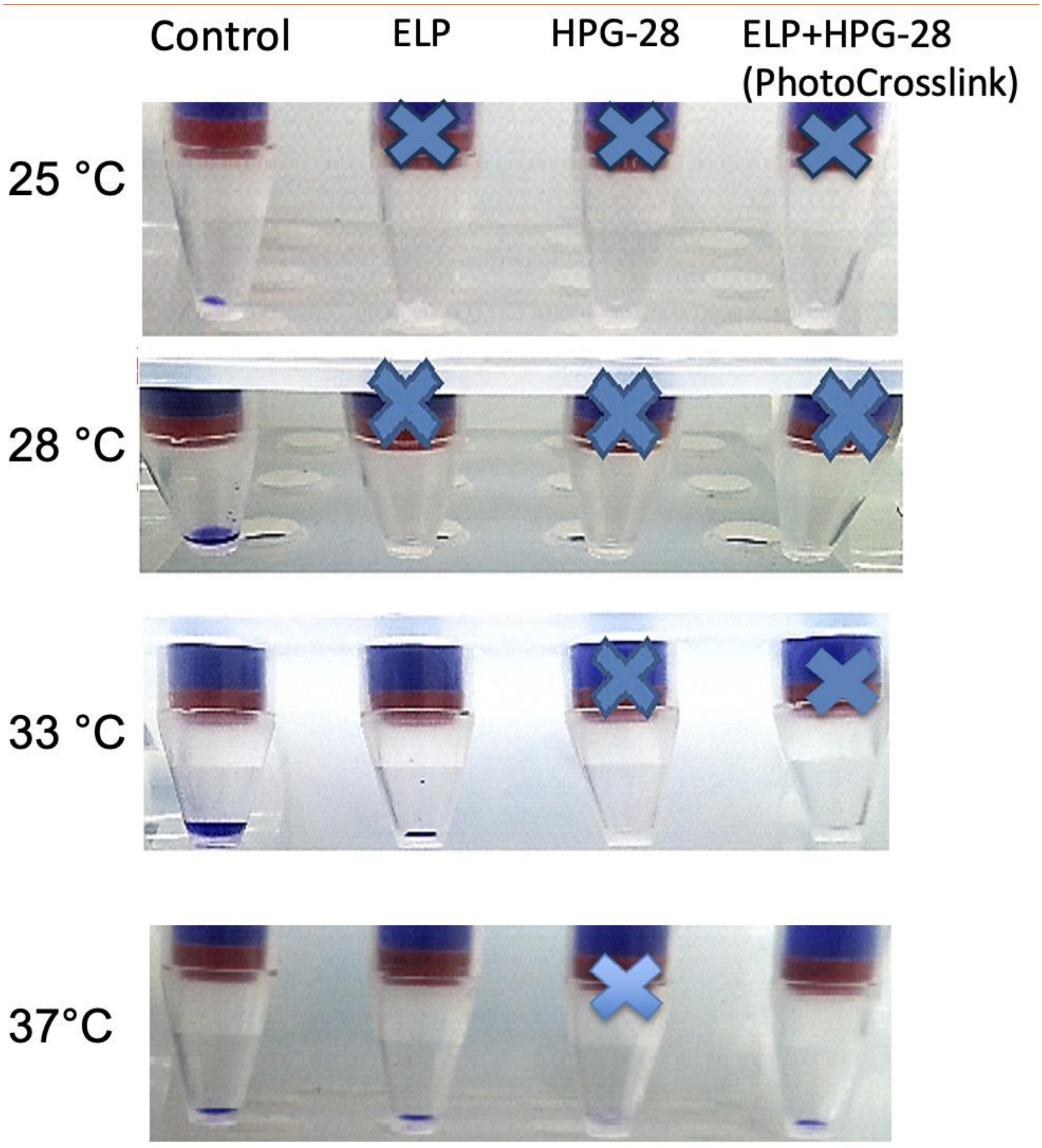

Figure S2. Permeation experiment of crystal violet. Photos of the solutions after filtration through YM-30 uncoated (control) or coated membranes with one of three surface treatments: ELP 4-80, HPG-28, or ELP with HPG-28. The permeation tests were evaluated at four different temperatures: 25 ^{o}C, 28 ^{o}C, 33 ^{o}C, and 37 ^{o}C.
